# ^18^F-FDG PET/CT improves diagnostic certainty in native and prosthetic valve Infective Endocarditis over the modified Duke Criteria

**DOI:** 10.1007/s12350-021-02689-5

**Published:** 2021-06-24

**Authors:** Christopher P. Primus, Thomas A Clay, Maria S. McCue, Kit Wong, Rakesh Uppal, Shirish Ambekar, Satya Das, Sanjeev Bhattacharyya, L. Ceri Davies, Simon Woldman, Leon J. Menezes

**Affiliations:** 1 Barts Heart Centre, St Bartholomew’s Hospital, London, UK; 2 Queen Mary, University of London, London, UK; 3 University College London, London, UK; 4 UCL Institute of Nuclear Medicine, London, UK

**Keywords:** Infective endocarditis, PET/CT, modified Duke Criteria, native, prosthetic

## Abstract

**Background:**

International guidance recognizes the shortcomings of the modified Duke Criteria (mDC) in diagnosing infective endocarditis (IE) when transoesophageal echocardiography (TOE) is equivocal. ^18^F-FDG PET/CT (PET) has proven benefit in prosthetic valve endocarditis (PVE), but is restricted to extracardiac manifestations in native disease (NVE). We investigated the incremental benefit of PET over the mDC in NVE.

**Methods:**

Dual-center retrospective study (2010-2018) of patients undergoing myocardial suppression PET for NVE and PVE. Cases were classified by mDC pre- and post-PET, and evaluated against discharge diagnosis. Receiver Operating Characteristic (ROC) analysis and net reclassification index (NRI) assessed diagnostic performance. Valve standardized uptake value (SUV) was recorded.

**Results:**

69/88 PET studies were evaluated across 668 patients. At discharge, 20/32 had confirmed NVE, 22/37 PVE, and 19/69 patients required surgery. PET accurately re-classified patients from possible, to definite or rejected (NRI: NVE 0.89; PVE 0.90), with significant incremental benefit in both NVE (AUC 0.883 vs 0.750) and PVE (0.877 vs 0.633). Sensitivity and specificity were 75% and 92% in NVE; 87% and 86% in PVE. Duration of antibiotics and C-reactive Protein level did not impact performance. No diagnostic SUV cut-off was identified.

**Conclusion:**

PET improves diagnostic certainty when combined with mDC in NVE and PVE.

**Supplementary Information:**

The online version contains supplementary material available at 10.1007/s12350-021-02689-5.

## Introduction

Infective endocarditis (IE) is classically viewed as a rare diagnosis, however its incidence has risen since the turn of the century, associated with a surge in cardiovascular intervention in an aging population.^1^ Diagnosis is based on the modified Duke Criteria (mDC), with patients classified as either definite, possible or rejected for IE based on the clinical, imaging and microbiological features present.^2^ Foremost among these are typical findings on echocardiography and blood cultures; absence of either of these makes a definite diagnosis of IE difficult to achieve. Despite advances in microbiological and imaging techniques, however, the mDC remain only 80% sensitive and specific, with around a quarter of patients misclassified as possible IE despite pathologically proven disease,^3^–^5^ even with the use of transoesophageal echocardiography (TOE).^6^,^7^ The use of multimodality cross-sectional imaging has therefore gained traction and is now recommended.^2^

The use of ^18^F-Fluorodeoxyglucose positron emission tomography with computed tomography (^18^F-FDG PET/CT, PET) to aid in the diagnosis of prosthetic valve endocarditis (PVE) has been increasingly reported with high sensitivity and specificity,^8^ especially when using myocardial suppression techniques.^9^–^13^ Adding focal tracer avidity around a prosthetic valve as a major criterion to the mDC reduces the number of “possible” cases in a cohort of IE patients, and thus improves the diagnostic utility of these criteria.^14^ The focus of these studies has been mainly limited to PVE^15^ and cardiac implantable electronic device (CIED) infection,^16^ where PET confers incremental benefit over TOE due to elimination of acoustic shielding. By comparison, the role of PET in native valve endocarditis (NVE) is less well studied. When used, it has been limited to identifying extracardiac manifestations in NVE, with low sensitivity and specificity at the valve level, and a failure to meaningfully reclassify patients to confirm or refute the diagnosis.^9^,^12^

Barts Heart Centre (BHC) was formed in May 2015 following a merger of the cardiology departments of Barts Health NHS Trust and University College London Hospitals (UCLH) NHS Trust. This merger made BHC the single cardiac surgery referral center for North Central and East London, and resulted in a significant increase in the number of IE cases seen. In line with the European Society of Cardiology (ESC) IE guidelines, this prompted formalization of the UCLH model to form an Endocarditis Team.^2^ In this context, and with ~ 150 referrals of possible IE per year we sought to investigate the incremental benefit of PET over the mDC in both PVE and NVE using our Endocarditis Team model.

## Methods

### Patients and Endocarditis Team

**Figure 1:**
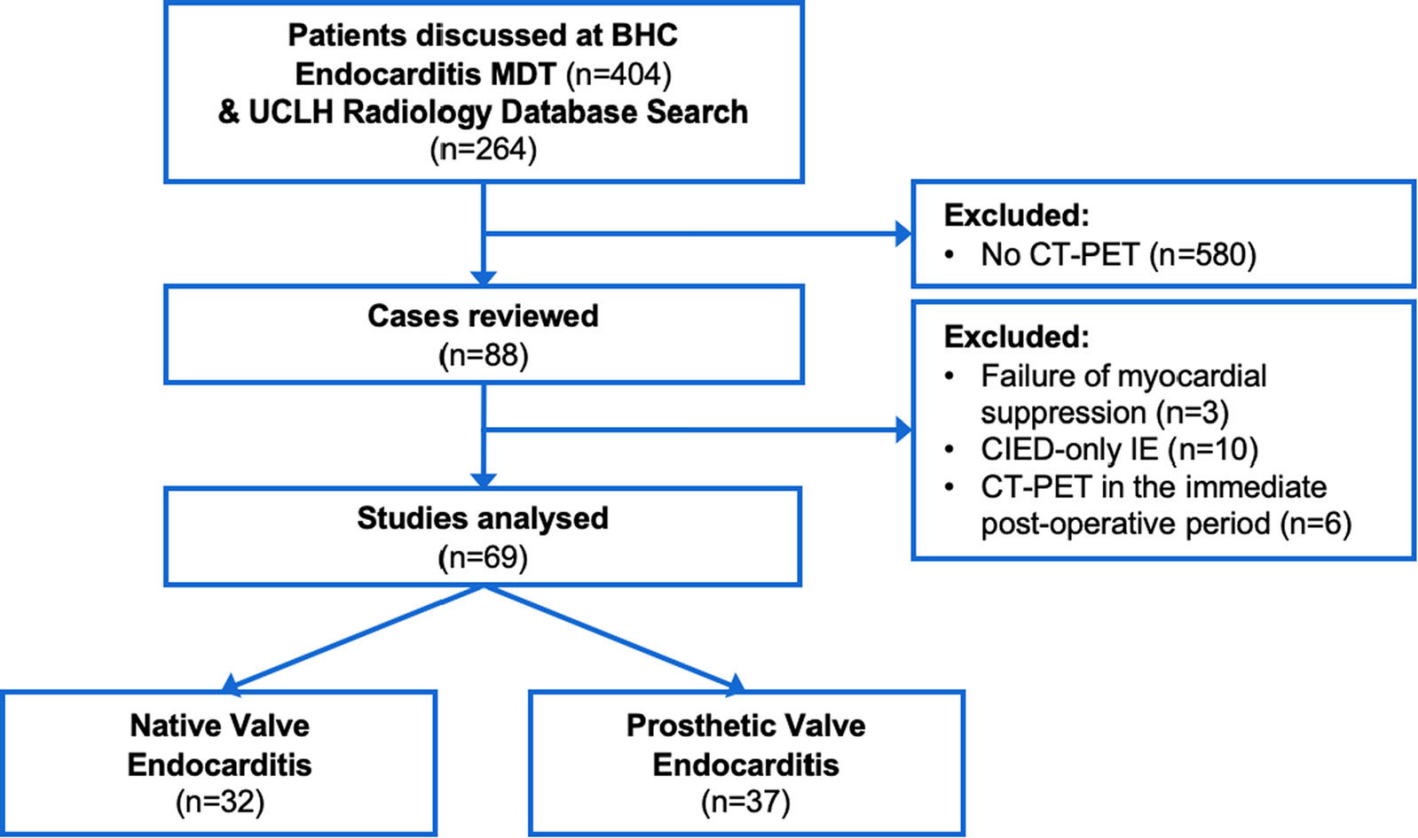
Consort diagram. All cases referred to the BHC Infective Endocarditis MDT (October 2015 to December 2018) and a search of the UCLH Radiology Database (January 2010 to October 2015) for ‘endocarditis’ and ‘PET’ were assessed for eligibility. Data were collected and categorized for NVE and PVE. *BHC*, Barts Heart Centre; *NVE*, native valve endocarditis; *PVE*, prosthetic valve endocarditis; *PET-CT*, ^18^Fluoride fluorodeoxyglucose position emission tomography with computed tomography; *UCLH*, University College London Hospitals

Under terms of an overarching audit, a dual center retrospective review identified all patients undergoing PET for IE from January 2010 to December 2018. Patients imaged early in the post-operative period following valve surgery for IE (< 3 months), those with CIED-only IE, and studies with failure of myocardial suppression were excluded (Figure 1).

Data were collected on all relevant clinical and imaging variables, particularly those pertinent to the mDC, duration of antibiotic therapy pre-PET and biochemical markers. Individual cases were scored using the mDC both pre- and post-PET by two independent investigators (CP & TC) in consensus. The definitive discharge diagnosis was recorded by surgical specimen in those who underwent operative intervention, or by Endocarditis Team consensus in those medically managed (excluding the PET findings).

The Endocarditis Team review all cases of IE referred to our Institution on a weekly basis. Prior to the formation of the BHC team, the core members led in the clinical care of patients with IE at UCLH via *ad hoc* bedside discussion.

### Image Acquisition of ^18^F-FDG PET-CT

All patients underwent myocardial suppression technique to suppress metabolic activity in the myocardium. This was achieved using a > 24-hour high fat, carbohydrate-restricted diet, a > 12-hour fast and intravenous injection of unfractionated heparin (50 IU/kg) 60 minutes prior to assessment with PET.^15^,^16^ PET-CT was only undertaken when blood glucose < 11 mmol/L as per local standard operating procedures. Following intravenous administration of ^18^F-FDG (4.5 MBq/kg) at a mean time of 64 minutes (SD 13 minutes) (mean activity 157 ± 39 MBq), we performed combined imaging with an MI DR PET-CT scanner (GE Healthcare, Chalfont St Giles, UK). An unenhanced, ungated CT was performed (64 detectors, at a pitch of 1.5 and a 2.5 mm collimation) from vertex to thigh for attenuation correction. A subsequent PET was performed at a bed overlap of 49% and a time per bed position of 100s. The reconstruction method was VUE Point FX, with 2 iterations, 12 subsets and a 5 mm Gaussian filter. All studies were standardized for display and reading with an SUV window threshold of 0-10^17^.

### Interpretation of ^18^F-FDG PET/CT

PET images were read in a blinded fashion by two independent investigators (CP & LM) with joint reading to resolve discrepancies in consensus, using attenuation and non-attenuation corrected images (the latter in particular for PVE). Myocardial suppression was graded as good, fair, poor or non-diagnostic,^17^ with non-diagnostic cases of unsuppressed diffuse myocardial uptake excluded from further analysis.

Studies were assessed for avidity of the culprit valve. The pattern and distribution of avidity was categorized as focal, heterogenous, homogenous or none (heterogenous and homogenous typically referred to as diffuse). An overall verdict (yes/no) was given on a case-by-case basis as to whether the PET was suspicious for IE or not, with a study considered positive if uptake was either ‘focal’ or ‘heterogenous’. Note was made if PET suggested an alternative diagnosis.

An elliptical region of interest (ROI) was placed over the valve, mediastinal blood pool and liver for semi-quantitative assessment of avidity using absolute mean and maximum standardized uptake values (SUV) allowing target-to-background analysis. In addition to avidity at the level of the cardiac valves, note was made of the presence of extracardiac uptake (including spleen, mediastinal lymph nodes, lungs, spine and sternum).

Studies were analyzed using the freely available Horos (version 3.3.5).

### Statistical Analysis

GraphPad Prism (version 7.0) and SPSS (version 25) were used for statistical analyses. Descriptive statistics were calculated for continuous variables, and *χ*^2^ for categorical data. Diagnostic performance was evaluated using Receiver Operating Characteristic (ROC) analysis and net reclassification index (NRI). Respective sensitivities, specificities, positive and negative predictive values for PET in both native and prosthetic valve disease were calculated using the discharge diagnoses, categorized as either confirmed or rejected IE as described above.

## Results

PET was undertaken in 88/668 patients, with 69 studies (10.3%) eligible for inclusion; 59/404 (14.6%) following the formalization of the Endocarditis Team in October 2015. The cohort featured 48 male patients, with an overall mean age of 61 years (range 21–89 years). Thirty-two (46%) were native valve patients and 37 cases had prosthetic valves, of which 20 were tissue and 17 mechanical prostheses. All patients, except one NVE patient, underwent assessment with TOE (patient refusal). Further baseline characteristics are described in Table 1.

**Table 1:** Cohort demographics

	All patients		Native valves		Prosthetic valves	
Patients	69		32	(46.4%)	37	(53.6%)
Male	48	(69.6%)	26	(81.3%)	22	(59.5%)
Bioprosthetic	–		–		20	(54%)
Mechanical	–		–		17	(46%)
Mean age (range)	61.0	(21–89)	60.0	(21–89)	60.8	(24–89)
Confirmed endocarditis at discharge	42	(60.1%)	20	(62.5%)	22	(59.5%)
Culprit valve						
Aortic	45	(65.2%)	13	(40.6%)	32	(86.5%)
Mitral	13	(18.8%)	9	(28.1%)	4	(10.8%)
Aortic and mitral	2	(2.9%)	2	(6.3%)	0	(0.0%)
Other	9	(8.7%)	8	(25.0%)	1	(2.7%)
Management strategy						
Medical	50	(72.5%)	23	(71.9%)	27	(73.0%)
Surgical	19	(27.5%)	9	(28.1%)	10	(27.0%)
TOE						
Initial	68	(98.6%)	31	(96.9%)	37	(100%)
Repeated pre-PET	32	(46.3%)	11	(34.3%)	21	(56.8%)
Blood cultures						
Positive	51	(73.9%)	23	(71.9%)	28	(75.7%)
*Staphylococcus aureus*	20	(29.0%)	13	(40.6%)	7	(18.9%)
Other *Staphylococci*	5	(7.2%)	4	(12.5%)	1	(2.7%)
*Streptococci*	10	(14.5%)	3	(9.4%)	7	(18.9%)
*Enterococci*	10	(14.5%)	2	(6.3%)	8	(21.6%)
Other	6	(8.7%)	1	(3.1%)	5	(13.5%)
Mean CRP (mg/L) at PET (SD)	39.9	(39.8)	38.6	(29.8)	39.3	(45.4)
Median days of antibiotics pre-PET (IQR)	19.0	(10.0–30.0)	20.6	(9.5–25.0)	17.0	(11.5–33.0)

*IQR*, interquartile range; *SD*, standard deviation; *TOE*, transoesophageal echocardiogram

At discharge, 20/32 (63%) had confirmed NVE and 22/37 (59%) confirmed PVE, giving a total prevalence for IE in our cohort of 61%. Nineteen (28%) patients required surgical intervention, 9 (28%) NVE and 10 (27%) PVE, with the remaining 50/69 cases managed medically, as per European Society of Cardiology (ESC) and British Society for Antimicrobial Chemotherapy (BSAC) guidance.^2^,^18^*Staphylococcus aureus* was isolated in 20/69 (29.0%) patients; 18/69 (26.1%) were peripheral blood culture-negative (BCNIE) (Table 1).

The median duration of IE-targeted antibiotic therapy pre-PET was 20.6 (IQR 9.5-25.0) days in 30/32 NVE cases and 17.0 (IQR 11.5-33.0) days in 33/37 PVE cases. Categorical analysis of median duration of antibiotics showed no significant impact on PET performance in NVE, PVE or overall (*P* > 0.10). Mean CRP at the time of PET was 38.6 (SD ± 29.8) mg/L in 20/32 NVE cases and 39.3 (SD ±4 5.4) mg/L in 29/37 PVE cases. Prolonged duration of antibiotic therapy was associated with a downward trend in CRP at time of PET (β = − 0.11, *r*^2^ = 0.004). There was no difference in PET performance with CRP < 40 and ≥ 40 in NVE, PVE or overall (*P* > 0.10).^12^

Over a median follow-up of 3.21years (IQR 1.75-3.96, 211.7 patient-years), there were 9 episodes of further IE in 8 patients, with 1 treatment failure in a medically managed patient unfit for surgery. Median IE free duration was 379 days in these individuals (range 28-1095 days). Of these episodes, 5 required surgical intervention during the index admission and four were managed medically. Independent review of mDC post-PET and discharge diagnosis made by the Endocarditis Team showed PET to have correctly confirmed or refuted IE in all cases.

**Figure 2:**
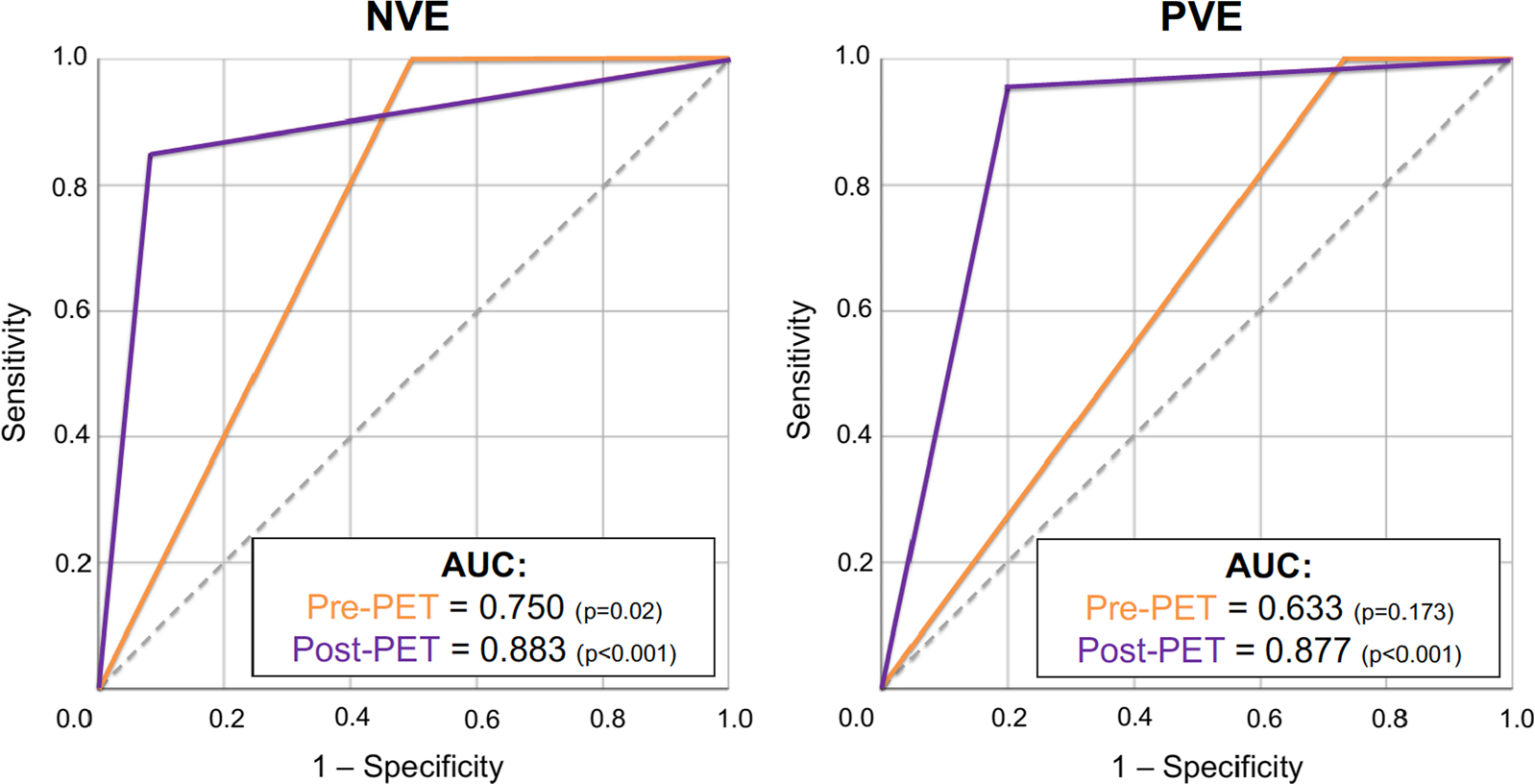
Incremental benefit of PET-CT with the modified Duke Criteria. ROC curves highlight significant incremental benefit of PET-CT over modified Duke Criteria alone in both PVE and NVE when compared to discharge diagnosis. *NVE*, native valve endocarditis; *PET-CT*, ^18^Fluoride fluorodeoxyglucose position emission tomography with computed tomography; *PVE*, prosthetic valve endocarditis; *ROC*, Receiver Operator Characteristic

Of the PET studies undertaken, 3/88 (3.4%) were excluded from analysis due to complete failure of myocardial suppression, rendering the scans non-diagnostic (Figure 1). All remaining scans eligible for inclusion were diagnostic, despite variable success in suppressing myocardial uptake (Table 2). Use of PET re-classified patients with possible IE as per mDC to either definite or rejected IE in both NVE and PVE, including the identification of an alternative, non-cardiac source of infection in 12 patients overall (16.9%). Quantification of this reclassification yielded NRI values of 0.89 for NVE and 0.90 for PVE (Table 3), where NRI > 0.5 suggests a beneficial test. ROC curves showed incremental benefit of PET over Duke’s criteria alone in both NVE (AUC 0.883 vs 0.750, *P* < 0.001) and PVE (AUC 0.877 vs 0.633, *P* < 0.001) compared to discharge diagnosis (Figure 2). PET sensitivity, specificity, positive and negative predictive values were 75%, 92%, 94% and 69% respectively in NVE, and 87%, 86%, 91% and 80% in PVE.

**Table 2:** Myocardial suppression and pattern of valve avidity

	Native valves (N = 32)	Prosthetic valves (N = 37)
Quality of myocardial suppression		
Good	23 (71.9%)	24 (64.9%)
Fair	4 (12.5%)	8 (21.6%)
Poor	5 (15.6%)	5 (13.5%)
Pattern of uptake at culprit valve		
Focal	16	18
Heterogenous	2	6
Homogenous	N/A	7
None	14	6

Studies were scored for success of myocardial suppression, and the pattern of uptake at the culprit valve. Background myocardial glucose uptake was suppressed with a high fat, carbohydrate-restricted diet, > 12-hour fast and intravenous injection of heparin immediately prior to assessment with ^18^F-FDG PET-CT. Three cases were excluded from analyses due to complete failure of myocardial suppression, rendering the studies non-diagnostic, and are not included in this table.

Focal or heterogenous uptake were considered suggestive of IE; homogenous uptake was suggestive of post-operative inflammation.

**Table 3:** Diagnostic reclassification following CT-PET

	Native valve endocarditis			Prosthetic valve endocarditis		
	Pre-PET	Post-PET	Discharge diagnosis	Pre-PET	Post-PET	Discharge diagnosis
Modified Duke Criteria						
Definite	14	16	20	14	21	22
Possible	12	2	–	19	3	–
Rejected	6	14	12	4	13	15

Net Reclassification Index		
Overall	0.89	0.90
Positive	0.44	0.50
Negative	0.45	0.40

PET reclassified patients to both confirm and refute IE, including by establishing a firm diagnosis in those with possible IE by modified Duke’s Criteria. It did so accurately, as confirmed by NRI. Positive NRI refers to correct identification of IE, and negative NRI refers to correctly refuting IE.

*IE*, Infective Endocarditis; *NRI*, Net Reclassification Index; *NVE*, native valve endocarditis; *PET-CT*, ^18^Fluoride fluorodeoxyglucose position emission tomography with computed tomography; *PVE*, prosthetic valve endocarditis

**Figure 3:**
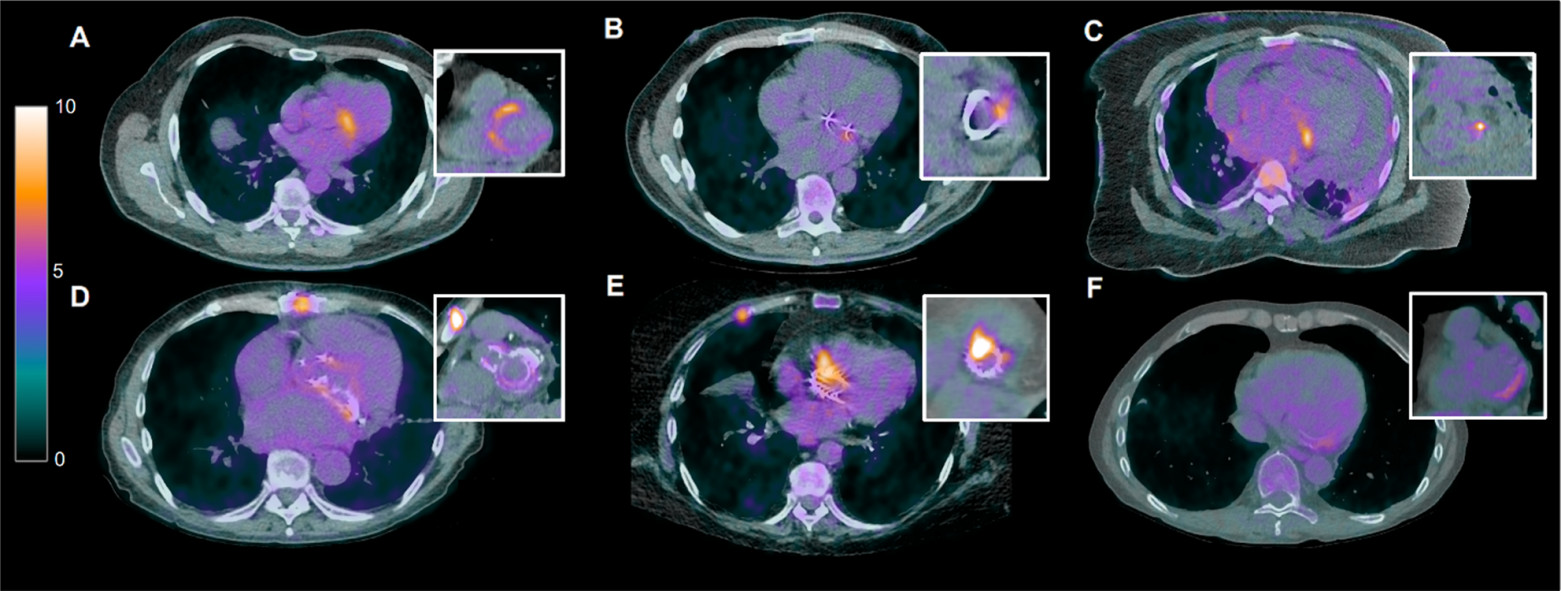
Representative images of Endocarditis on PET-CT. Images acquired following a high fat, low carbohydrate diet, 12-hour fast and intravenous heparin to achieve suppression of physiological myocardial uptake (myocardial suppression). Panel **A** NVE of mitral valve; Panel **B** PVE of a mitral valve ring; Panel **C** NVE of mitral valve with pericardial effusion; Panel **D** post-surgical uptake in mechanical aortic and mitral valve replacements; Panel **E** PVE with root abscess in TAVI; Panel **F** NVE of mitral valve. *NVE*, native valve endocarditis; *PET-CT*, ^18^Fluoride fluorodeoxyglucose position emission tomography with computed tomography; *PVE*, prosthetic valve endocarditis; *TAVI*, transcatheter aortic valve implant

Focal uptake was observed in 16 (50%) cases of NVE and 18 (49%) in PVE (Table 2). Diffuse uptake was seen in 13 cases (35%) of PVE; 4 were true positive IE with heterogenous uptake, and 7 true negative with homogenous uptake representing post-operative change in the latter category. Illustrative examples are depicted in Figure 3. Embolic phenomena were identified in 14 (20%) patients; splenic avidity and low volume mediastinal lymphadenopathy were not considered here, as while suggestive of infection, they are not specific for IE.^19^ Semi-quantitative analysis using SUVs showed no clear diagnostic cut-off for SUV, SUV_max_ or SUV_mean_, nor when these parameters were normalized to hepatic and mediastinal blood pool uptake (Table 4).

**Table 4:** Semi-quantitative analysis of PET-CT valve avidity using standardized uptake values

	Native valve endocarditis (N = 21)		Prosthetic valve endocarditis (N = 32)	
	Median	IQR	Median	IQR
SUV_max_				
Confirmed IE	3.36	2.78–5.74	4.78	3.78–6.20
Rejected IE	2.62	2.00–3.24	3.88	2.14–7.17
SUV_mean_				
Confirmed IE	1.92	1.80–2.06	2.48	2.13–2.85
Rejected IE	1.93	1.48–2.16	2.29	1.59–3.10

	AUC	SE	AUC	SE
Ratio of SUV_max_				
Mediastinal blood pool	0.55	0.13	0.61	0.09
Liver	0.52	0.13	0.51	0.09
Ratio of SUV_mean_				
Mediastinal blood pool	0.61	0.13	0.59	0.10
Liver	0.60	0.12	0.50	0.09

ROIs were identified at the culprit valve, mediastinal blood pool and liver. SUV_mean_ and SUV_max_ was obtained and ratios calculated. No optimal value to confirm or refute IE was identified across all indices (*P* > 0.10 by Student t-test and ROC). Data presented as median (IQR) for valve ROI and AUC (SE) for standardized values.

*AUC*, area under the curve; *IQR*, interquartile range; *PET-CT*, ^18^Fluoride fluorodeoxyglucose position emission tomography with computed tomography; *ROI*, region of interest; *SE*, standard error; *SUV*, standardized uptake value

## Discussion

The climbing incidence of IE, and clear benefits of the Endocarditis Team mandate a guideline-driven approach to the diagnosis of IE.^2^ The benefit of multimodality imaging is clearly recognized in the current era, with PET having a role where the likelihood of IE is high but TOE remains equivocal. In NVE, this has focussed on the identification of extracardiac emboli.^20^,^21^ The recent results of the EURObservational Research Programme of the ESC Endocarditis Registry (EORP EuroENDO Registry, EuroENDO) highlight the increasing use of PET at 16.6%,^8^ but this has mainly focussed on PVE and CIED-IE.^12^,^22^ The increasing utilization of PET is reflected in the current study, where PET usage increased from 3.8% (January 2010-September 2015) to 14.6% (October 2015-December 2018) in line with the most recent ESC guidelines and the increasing body of evidence for PET in IE.^2^,^17^

IE remains a diagnostic challenge, especially in the absence of positive major criterion of the mDC. In our cohort, all patients had ongoing clinical suspicion of IE following equivocal TOE in 68/69 cases (N = 1, patient refusal), and 26% with likely BCNIE (Table 1). NRI highlights that PET accurately re-classifies individual patients with IE in both NVE (NRI 0.89) and PVE (NRI 0.90) (Table 3), as well as overall by ROC analysis (Figure 2). PET has been used with high specificity in NVE in previous studies,^12^,^23^ predominantly through identification of septic emboli.^17^ Critically, and uniquely to this study, however, NRI highlights the ability of PET to reliably confirm (positive reclassification) and refute (negative reclassification) the diagnosis (positive and negative NRI 0.40-0.50) through evaluation at valve level alone (Table 3).

Our data add further weight to the growing body of evidence advocating the sensitivity and specificity of PET in PVE, and corroborate our ability to utilize PET appropriately in IE. However, we have now been able to additionally demonstrate comparable sensitivity and specificity in NVE, at 75% and 92%, respectively, as with one similar series to date.^23^ Furthermore, when PET is used in this manner, we see the incremental benefit over the mDC in both NVE and PVE (AUC 0.883 NVE, AUC 0.877 PVE) with a prevalence of confirmed IE in 61% of our cohort at discharge (Figure 2). This is in comparison to similarly-sized series in the literature, with a sensitivity of 17.5%-57% in NVE,^9^,^12^,^19^,^20^,^24^,^25^ and only 28.0% in EuroENDO.^8^

Given the lack of typical TOE findings of significant valvular insufficiency and/or presence of vegetations in this cohort, it is unsurprising that fifty patients (72%) lacked a surgical indication and were managed medically. This is despite 99% of patients having at least one TOE, a high rate compared to 81%-88% of patients in other studies. 12,13 BCNIE also drives the use of PET in IE, as a lack of positive cultures also makes diagnosis by mDC challenging, with rates ranging from 10%-46% in other series.^8^,^12^,^13^,^19^ However, correctly diagnosing IE is critical to ensure appropriate inpatient treatment and outpatient monitoring. Follow-up of these patients shows agreement with discharge diagnosis derived by the Endocarditis Team (without use of the PET data reported here) in all patients, with only 3 patients re-presenting with IE within 24 months, over 212 patient-years of follow-up.

Identifying the factors responsible for the high performance of PET in the current cohort is critical. Factors thought responsible for the low sensitivity of PET in NVE are well summarized in the literature, from both technical limitations of the modality as well as the pathophysiology of NVE.^12^,^13^,^17^,^23^ These include discrete vegetations with less common paravalvular complications in NVE, a less aggressive local inflammatory response compared to PVE resulting in lower FDG uptake, duration of antibiotics pre-PET and CRP at time of PET.

Achieving adequate myocardial suppression is imperative to the successful use of PET in IE, with ~ 85% of suppression graded as good or fair in our cohort, and only 3/72 (4%) studies found to be non-diagnostic and therefore excluded from analysis. Even when myocardial suppression was poor, meaningful valvular assessment was still possible in this cohort based on visual analysis. This is in comparison to failure of myocardial suppression in 5%-32% of the cohort in similar studies of NVE and PVE.^12^,^13^,^23^

In our practice, PET is used where the diagnosis remains unclear despite 99% utilization of high-quality TOE (with lower rates in other series), including in patients with NVE. This is particularly important as this group of patients typically lack valve findings that mandate early surgery, hence diagnostic equipoise. Our approach is to therefore repeat echocardiography prior to PET, in order to easily identify those in whom valve dysfunction may have developed. However, this is reflected in the long median duration of antibiotics (19 days, IQR 10-30 days) prior to PET and downward trend in CRP (mean 39.9 mg/L, SD 39.8 mg/L) when compared to other studies.^12^,^13^,^19^,^23^ Our data suggest that when PET is targeted to those patients in whom the possibility of IE is high, but the mDC are equivocal, PET can add meaningful information irrespective of inflammatory marker levels or duration of antibiotic therapy.^12^ This finding may be explained by the pathophysiology of the disease; the long time-to-PET in the present study may allow for sufficient valvular inflammation to develop meeting the threshold for detection using ^18^F-FDG.^17^ This is however in contrast to a previous study where a lack of inflammatory response, or suppression of that response with antibiotics, has been associated with poor sensitivity of PET in NVE, with a proposed CRP cut-off of 40 mg/L.^13^ This relative delay to PET reflects real-world practice of a high-throughput nuclear medicine department in the United Kingdom’s National Health Service, in comparison to other series’ where PET was typically undertaken in < 10 days.^8^,^12^,^13^,^19^,^23^ with not all patients receiving broad-spectrum antibiotics for possible IE.^23^

We would suggest the use of PET in IE requires involvement of an Endocarditis Team with high volume throughput. This will both optimize case selection and improve technical reading of the study. This is particularly important to correctly distinguish valve from myocardial tracer uptake, and recognize patterns of uptake typical and atypical for IE, especially following previous cardiac surgery (Figure 3). Our analyses did not identify a clear semi-quantitative indicator to confirm or refute IE based on SUV, though this has been posited in other studies.^13^,^20^ In our experience, this failure to identify a semi-quantitative cut-off is a consequence of often low-grade focal avidity in NVE even with good myocardial suppression, and diffuse patchy uptake with a high standard deviation of SUV in PVE. Alternative analyses, such as doughnut-shaped ROIs or machine learning, or scanning techniques, such as ECG- and/or respiratory-gating may yet highlight a computed cut-off suggestive of valve infection.

The results of this study suggest that the real-world application of PET to patients with IE has meaningful benefit. Nonetheless, a formal prospective multicenter diagnostic accuracy study with hard endpoints is warranted, and we would argue should include patients with both NVE and PVE.

### Limitations

Advances in IE have been hindered by low incidence and a lack of randomized trials as a result.^26^ This retrospective analysis is based on real-world application of PET in a cohort of ‘all-comers’ referred to an Endocarditis Team discussing approximately 150 cases per annum, and therefore mitigates patient selection bias. Furthermore, our approach to multimodality imaging in patients with possible IE has been driven by experience in the context of international guidance, with a fixed pathway for PET utilization in both NVE and PVE as described earlier in the manuscript. Despite the high throughput of our center, utilization rates are similar to those seen in the current EuroENDO registry,^8^ and explains the limited patient cohort presented here. While similar in size to other studies in the literature, this study adds a unique perspective in NVE and adds to the growing body of evidence for the use of PET in IE.

The incidence of IE and the limitations of the mDC may cause bias when relying on expert consensus to confirm or refute IE in medically managed patients, and is a significant issue in all IE studies without surgical specimens. However, this is where the guidance currently supports the use of PET, where the diagnosis is not clear, and therefore surgical intervention is not necessarily mandated. Blinded scoring of the mDC and imaging analysis by the research team reduce the limitations of expert consensus in our study, and is supported by net reclassification following PET, highlighting the benefit of PET overall and for reclassification of individual patients. This impact is further mitigated by follow-up data that suggest the correct diagnosis was made, especially given a low incidence of recurrent episodes of IE over 212 patient-years.

Despite these limitations, the incremental benefit of PET in both NVE and PVE described herein suggests meaningful benefit. However, we would only advocate the routine use of PET where the diagnosis is equivocal after high-quality TOE and surgery is not mandated for another indication.

## Conclusions

In this retrospective analysis, we highlight the incremental benefit of PET for the diagnosis of IE in both native and prosthetic disease. PET performs well irrespective of inflammatory markers or duration of IE-focussed antibiotic treatment. We advocate the use of PET by expert Endocarditis Teams where both NVE and PVE is suspected, but TOE remains equivocal.

## New Knowledge Gained

The literature consistently identify poor sensitivity of PET/CT for NVE. We highlight that in a high-volume center, PET can be used to contribute to the diagnosis of both NVE and PVE in a meaningful manner. PET provides meaningful information at valve level in PVE and NVE, to help confirm and refute the diagnosis (NRI), outperforms mDC alone (AUC) and has higher than reported sensitivity in NVE. We further explore duration of antibiotics, CRP at time of PET and time to PET to explain why our findings differ to the rest of the literature.

## Supplementary Information

Below is the link to the electronic supplementary material.

Supplementary file1 (PPTX 3408 kb)

Supplementary file2 (PPTX 3221 kb)

## Abbreviations

BCNIE: Blood culture-negative infective endocarditis
ESC: European Society of Cardiology
IE: Infective endocarditis
mDC: Modified Duke Criteria
NRI: Net reclassification index
NVE: Native valve endocarditis
PET-CT: ^18^F-Fluorodeoxyglucose positron emission tomography with computed tomography
PVE: Prosthetic valve endocarditis
SUV: Standardized uptake values
TOE: Transoesophageal echocardiogram
