# Supplementary material for: 18F-FDG PET/CT improves diagnostic certainty in native and prosthetic valve Infective Endocarditis over the modified Duke Criteria
Source: J Nucl Cardiol. 2021 Jun 24;29(5):2119–28. doi: 10.1007/s12350-021-02689-5 (PMC9553763; doi:10.1007/s12350-021-02689-5)
Supplement: Supplementary file 1 — Supplementary file1 (PPTX 3408 kb) [file 12350_2021_2689_MOESM1_ESM.pptx]

## Slide 1
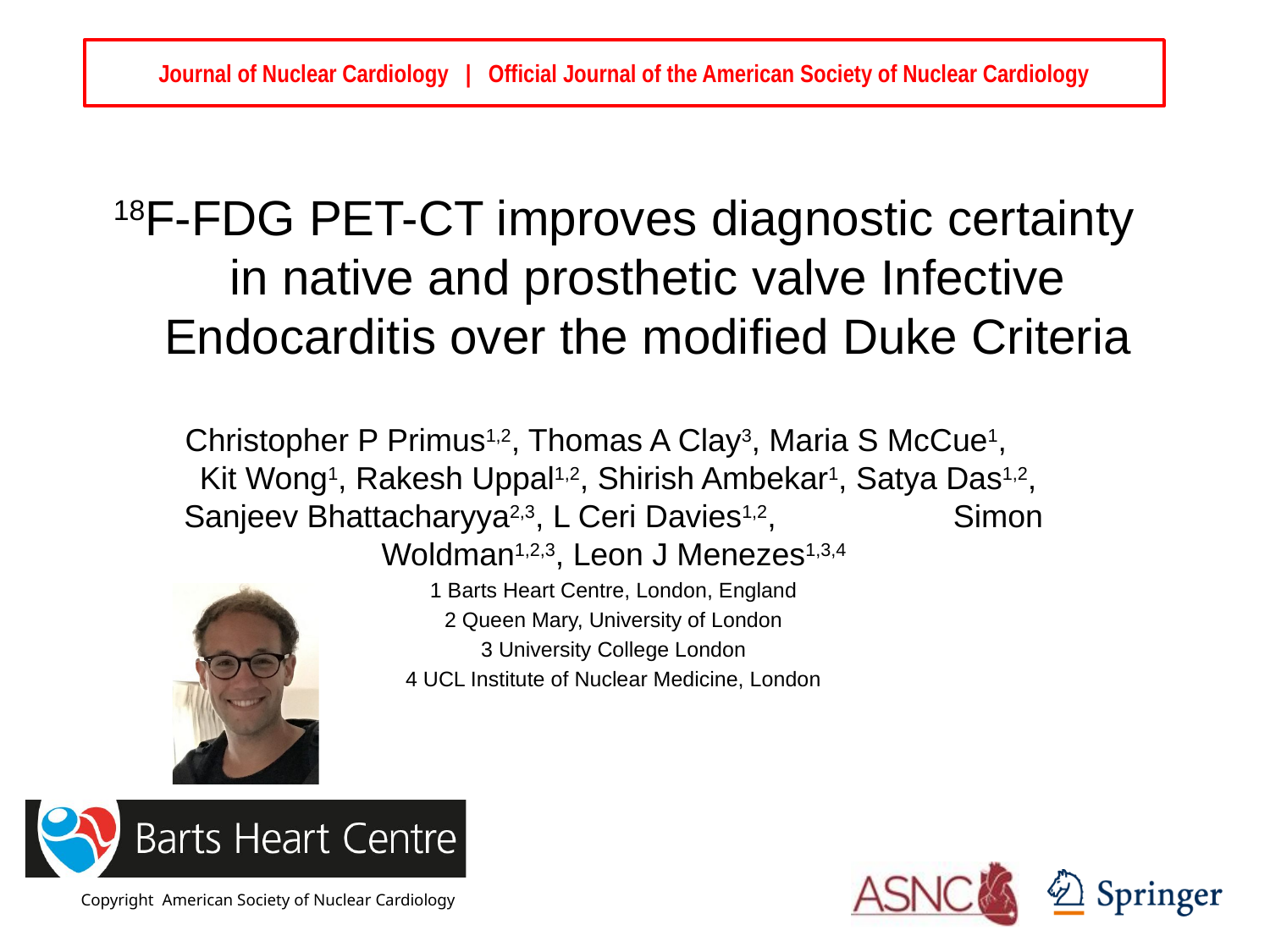

Journal of Nuclear Cardiology | Official Journal of the American Society of Nuclear Cardiology
# 18F-FDG PET-CT improves diagnostic certainty in native and prosthetic valve Infective Endocarditis over the modified Duke Criteria
Christopher P Primus1,2, Thomas A Clay3, Maria S McCue1, Kit Wong1, Rakesh Uppal1,2, Shirish Ambekar1, Satya Das1,2, Sanjeev Bhattacharyya2,3, L Ceri Davies1,2, Simon Woldman1,2,3, Leon J Menezes1,3,4
1 Barts Heart Centre, London, England
2 Queen Mary, University of London
3 University College London
4 UCL Institute of Nuclear Medicine, London
Copyright American Society of Nuclear Cardiology

## Slide 2
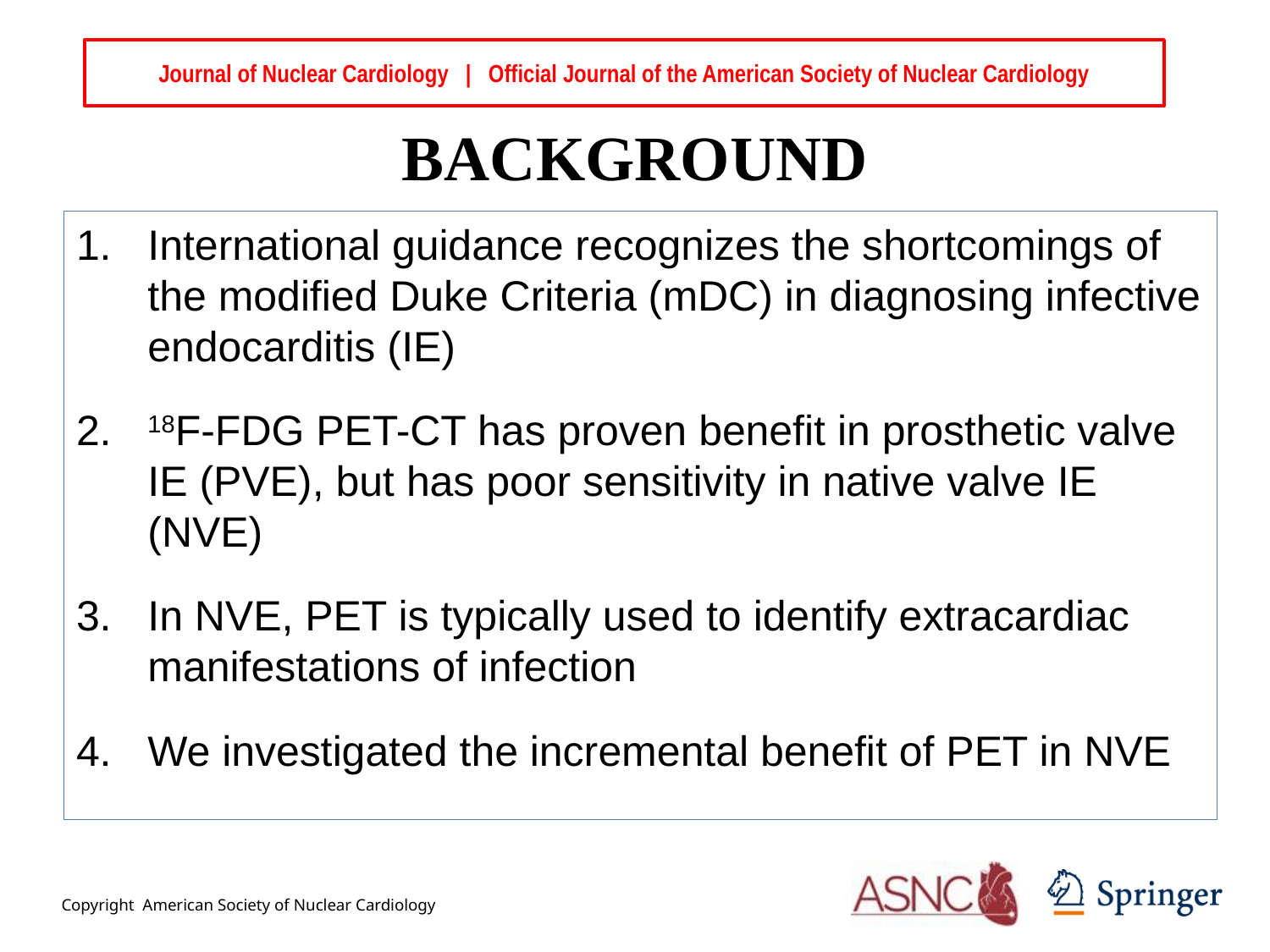

Journal of Nuclear Cardiology | Official Journal of the American Society of Nuclear Cardiology
# BACKGROUND
International guidance recognizes the shortcomings of the modified Duke Criteria (mDC) in diagnosing infective endocarditis (IE)
18F-FDG PET-CT has proven benefit in prosthetic valve IE (PVE), but has poor sensitivity in native valve IE (NVE)
In NVE, PET is typically used to identify extracardiac manifestations of infection
We investigated the incremental benefit of PET in NVE
Copyright American Society of Nuclear Cardiology

## Slide 3
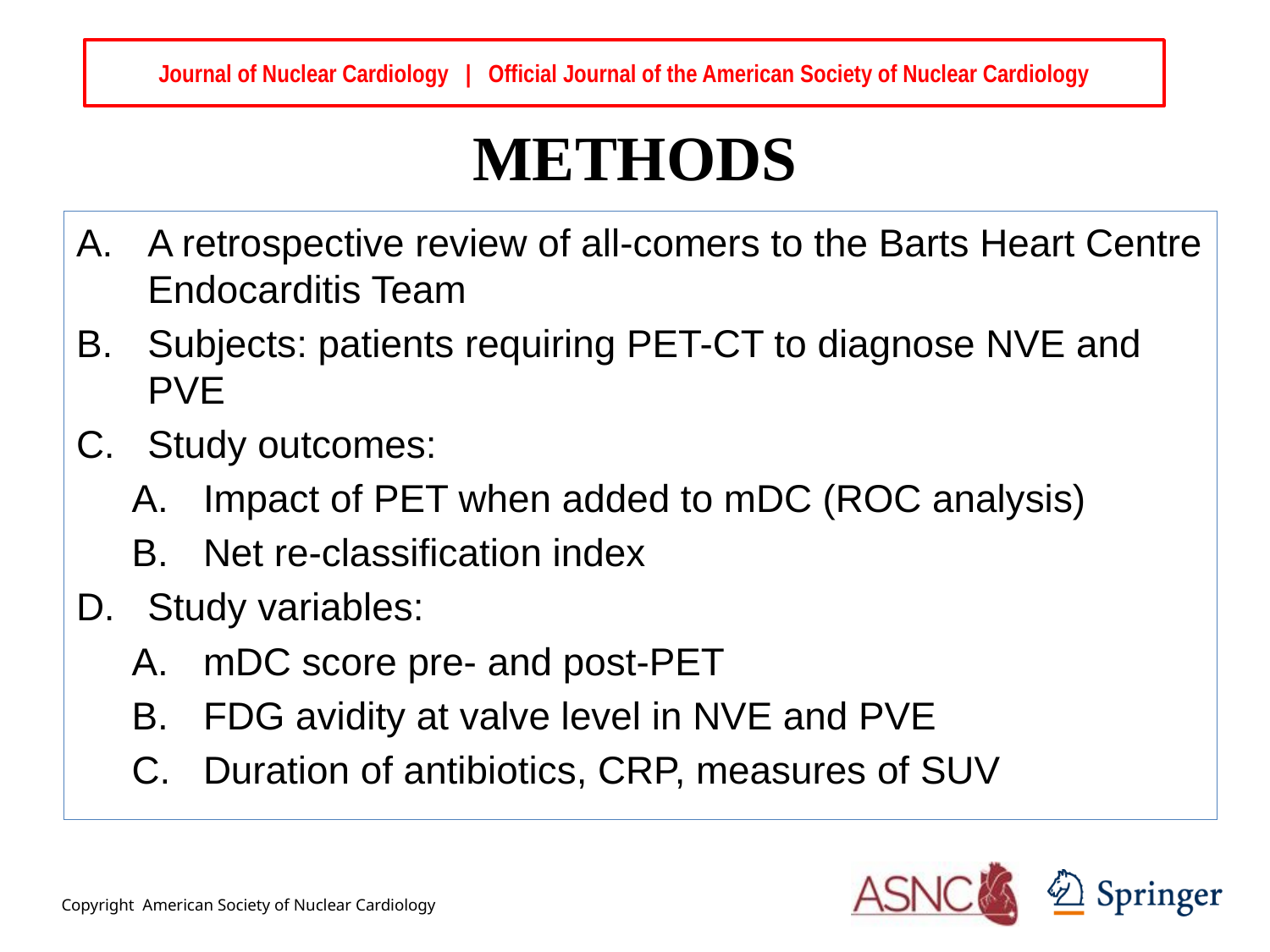

Journal of Nuclear Cardiology | Official Journal of the American Society of Nuclear Cardiology
# METHODS
A retrospective review of all-comers to the Barts Heart Centre Endocarditis Team
Subjects: patients requiring PET-CT to diagnose NVE and PVE
Study outcomes:
Impact of PET when added to mDC (ROC analysis)
Net re-classification index
Study variables:
mDC score pre- and post-PET
FDG avidity at valve level in NVE and PVE
Duration of antibiotics, CRP, measures of SUV
Copyright American Society of Nuclear Cardiology

## Slide 4
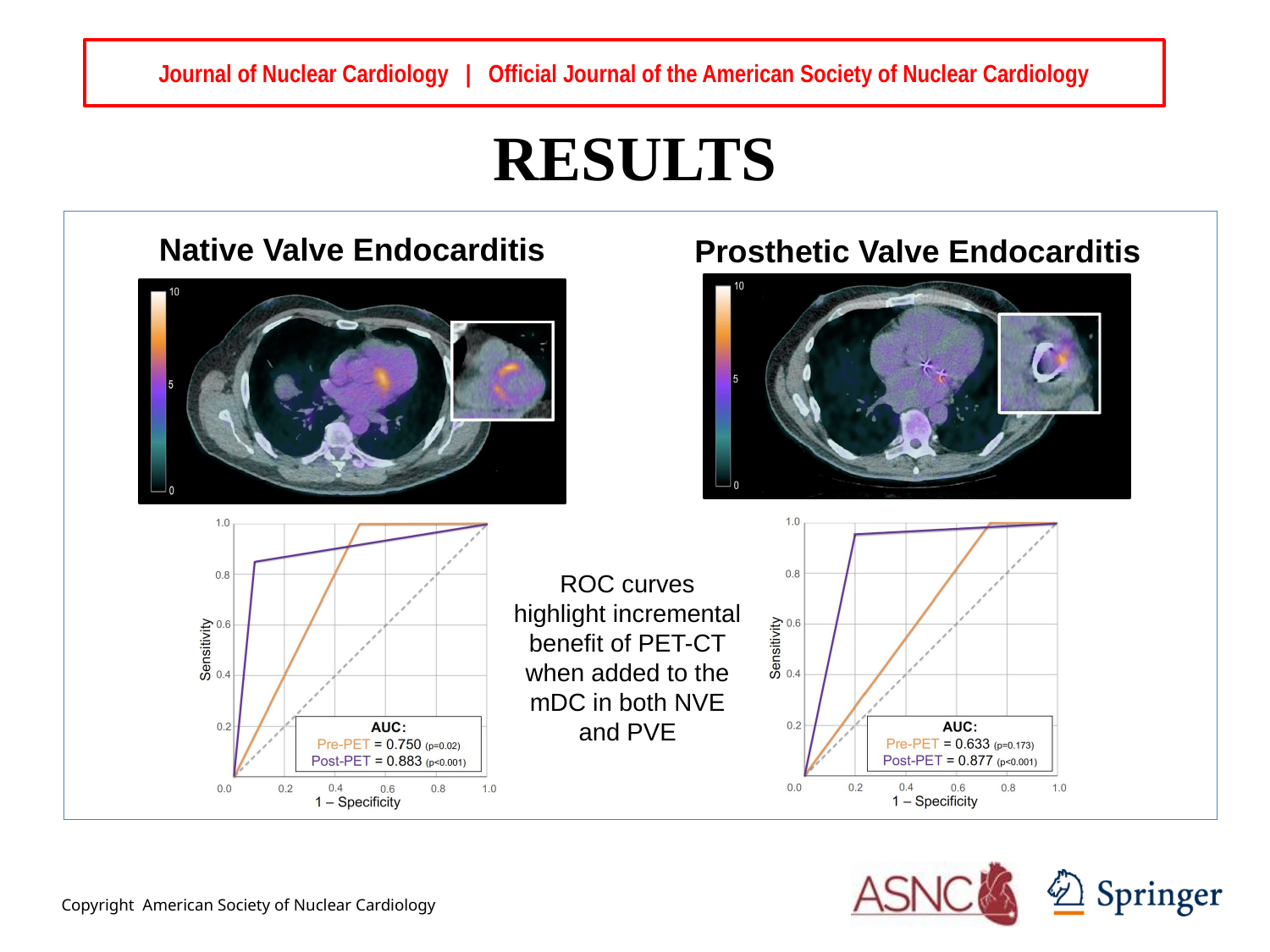

Journal of Nuclear Cardiology | Official Journal of the American Society of Nuclear Cardiology
# RESULTS
Native Valve Endocarditis
Prosthetic Valve Endocarditis
ROC curves highlight incremental benefit of PET-CT when added to the mDC in both NVE and PVE
Copyright American Society of Nuclear Cardiology

## Slide 5
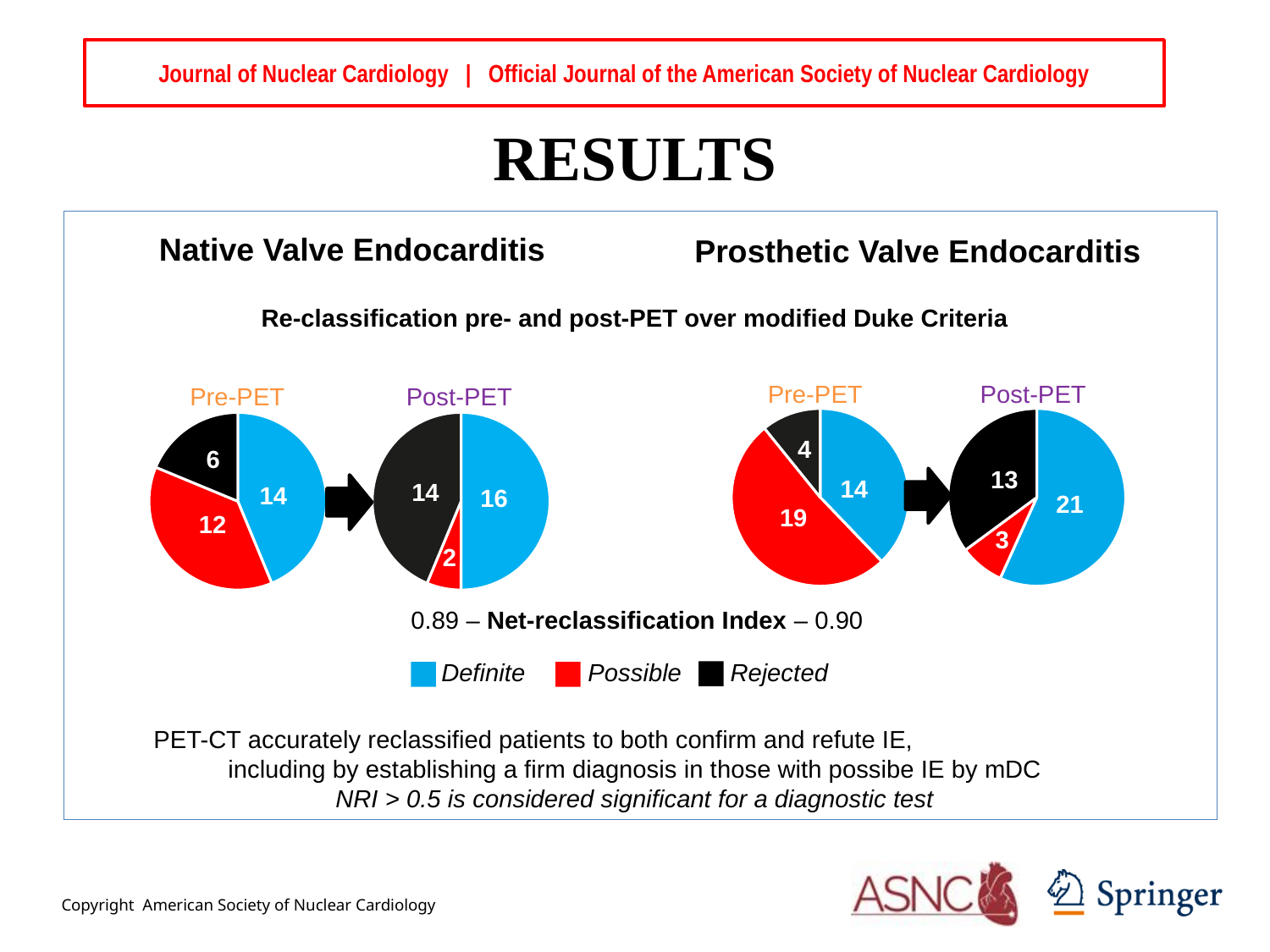

Journal of Nuclear Cardiology | Official Journal of the American Society of Nuclear Cardiology
# RESULTS
Native Valve Endocarditis
Prosthetic Valve Endocarditis
Re-classification pre- and post-PET over modified Duke Criteria
Pre-PET
Post-PET
### Chart
| Category | Column1 |
|---|---|
| Definite | 14.0 |
| Possible | 19.0 |
| Rejected | 4.0 |
### Chart
| Category | Sales |
|---|---|
| Definite | 21.0 |
| Possible | 3.0 |
| Rejected | 13.0 |13
14
21
19
3
Pre-PET
Post-PET
### Chart
| Category | Column1 |
|---|---|
| Definite | 16.0 |
| Possible | 2.0 |
| Rejected | 14.0 |
### Chart
| Category | Column1 |
|---|---|
| Definite | 14.0 |
| Possible | 12.0 |
| Rejected | 6.0 |14
14
16
12
2
0.89 – Net-reclassification Index – 0.90
Definite Possible Rejected
PET-CT accurately reclassified patients to both confirm and refute IE, including by establishing a firm diagnosis in those with possibe IE by mDC
NRI > 0.5 is considered significant for a diagnostic test
Copyright American Society of Nuclear Cardiology

## Slide 6
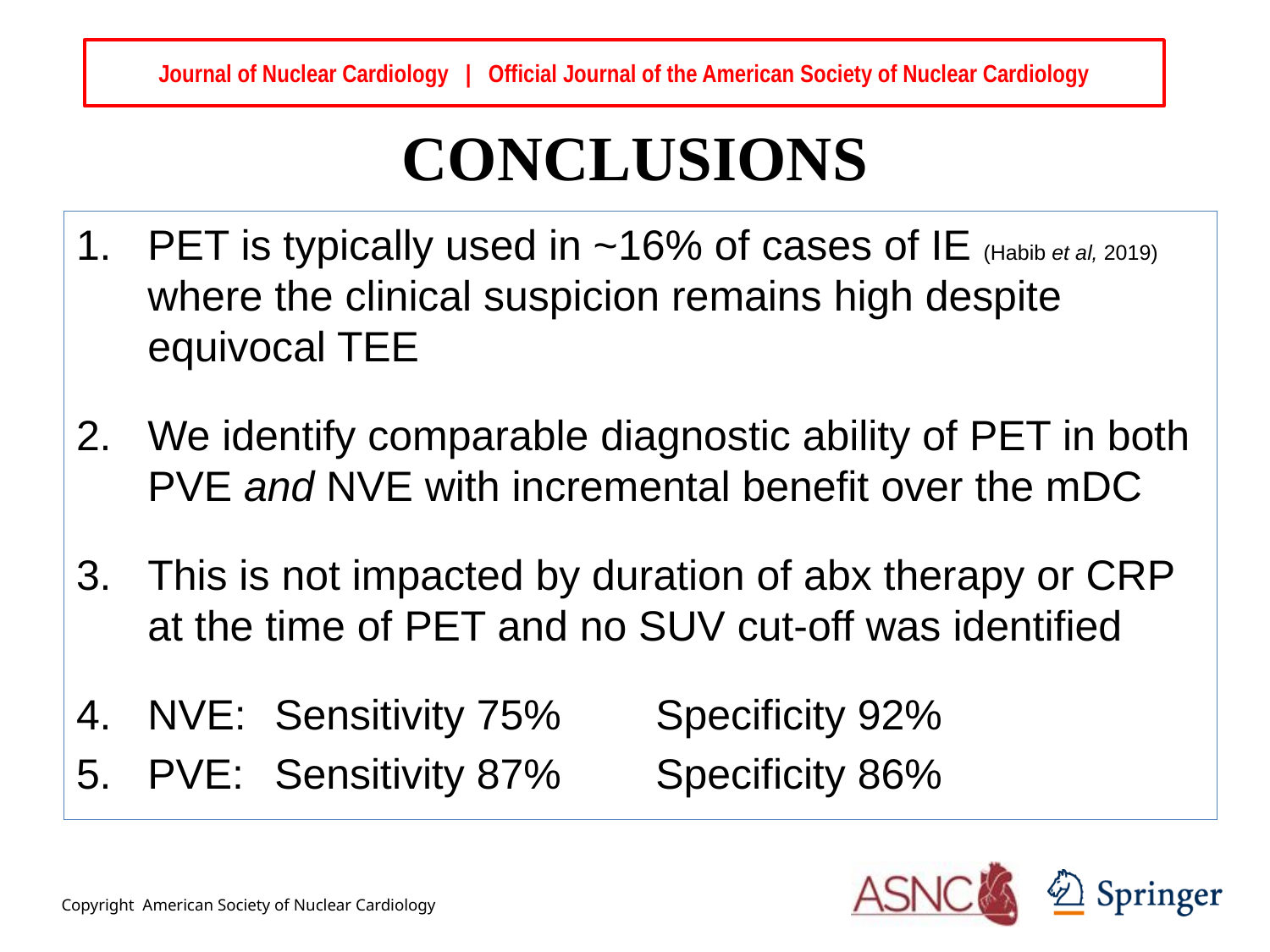

Journal of Nuclear Cardiology | Official Journal of the American Society of Nuclear Cardiology
# CONCLUSIONS
PET is typically used in ~16% of cases of IE (Habib et al, 2019) where the clinical suspicion remains high despite equivocal TEE
We identify comparable diagnostic ability of PET in both PVE and NVE with incremental benefit over the mDC
This is not impacted by duration of abx therapy or CRP at the time of PET and no SUV cut-off was identified
NVE: 	Sensitivity 75%	Specificity 92%
PVE:	Sensitivity 87%	Specificity 86%
Copyright American Society of Nuclear Cardiology
