# Supplementary material for: 18F-FDG PET/CT improves diagnostic certainty in native and prosthetic valve Infective Endocarditis over the modified Duke Criteria
Source: J Nucl Cardiol. 2021 Jun 24;29(5):2119–28. doi: 10.1007/s12350-021-02689-5 (PMC9553763; doi:10.1007/s12350-021-02689-5)
Supplement: Supplementary file 2 — Supplementary file2 (PPTX 3221 kb) [file 12350_2021_2689_MOESM2_ESM.pptx]

## Slide 1
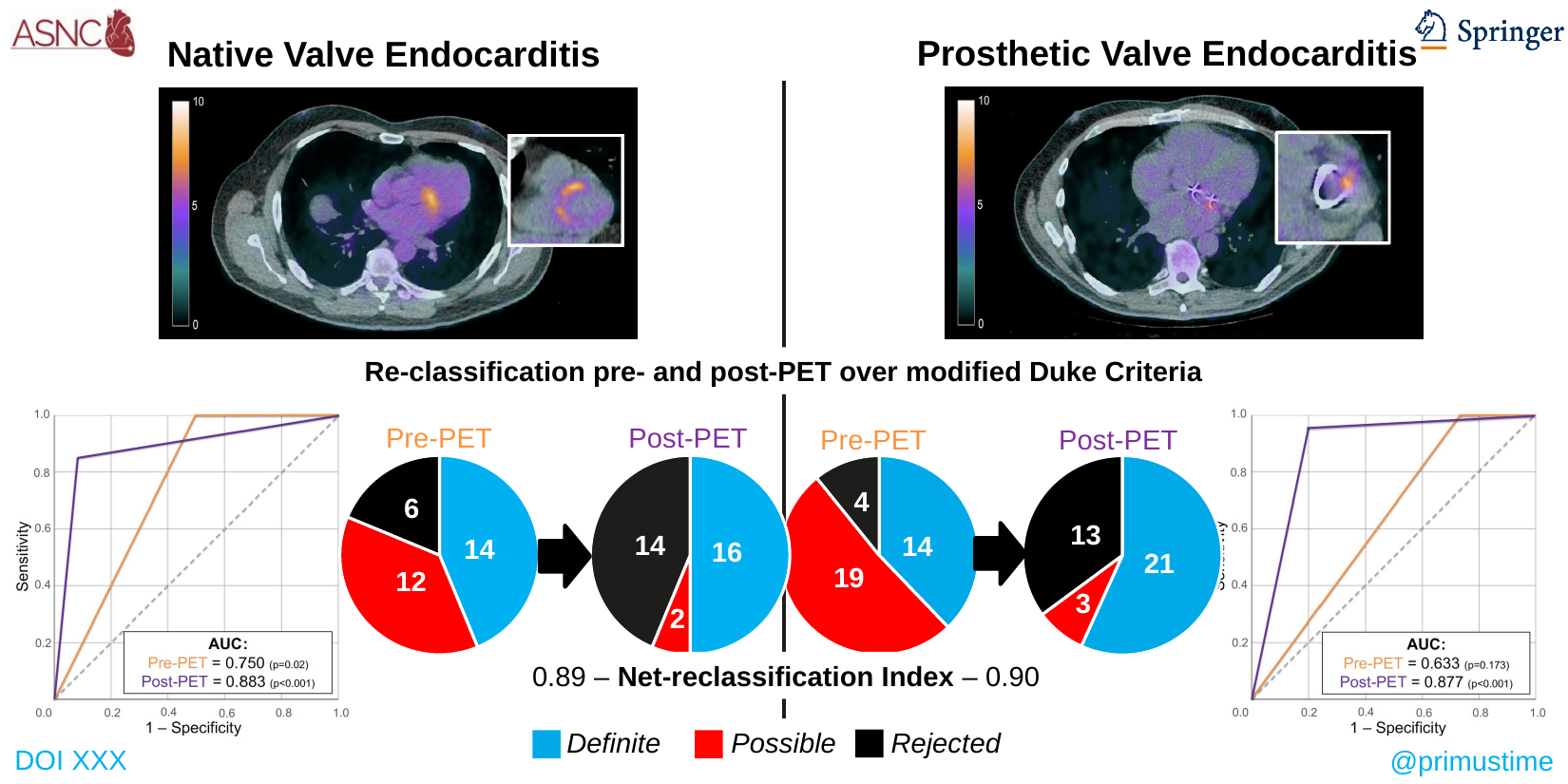

Prosthetic Valve Endocarditis
Native Valve Endocarditis
Re-classification pre- and post-PET over modified Duke Criteria
Pre-PET
Post-PET
### Chart
| Category | Column1 |
|---|---|
| Definite | 16.0 |
| Possible | 2.0 |
| Rejected | 14.0 |
### Chart
| Category | Column1 |
|---|---|
| Definite | 14.0 |
| Possible | 12.0 |
| Rejected | 6.0 |14
14
16
12
2
Pre-PET
Post-PET
### Chart
| Category | Column1 |
|---|---|
| Definite | 14.0 |
| Possible | 19.0 |
| Rejected | 4.0 |
### Chart
| Category | Sales |
|---|---|
| Definite | 21.0 |
| Possible | 3.0 |
| Rejected | 13.0 |13
14
21
19
3
0.89 – Net-reclassification Index – 0.90
Definite Possible Rejected
DOI XXX
@primustime
